# Novel Exopolysaccharide from Marine *Bacillus subtilis* with Broad Potential Biological Activities: Insights into Antioxidant, Anti-Inflammatory, Cytotoxicity, and Anti-Alzheimer Activity

**DOI:** 10.3390/metabo12080715

**Published:** 2022-07-31

**Authors:** Basel A. Abdel-Wahab, Hanaa F. Abd El-Kareem, Ahmad Alzamami, Cinderella A. Fahmy, Basem H. Elesawy, Maged Mostafa Mahmoud, Ahmed Ghareeb, Ahmad El Askary, Hebatallah H. Abo Nahas, Nashwah G. M. Attallah, Najla Altwaijry, Essa M. Saied

**Affiliations:** 1 Department of Medical Pharmacology, College of Medicine, Assiut University, Assiut 7111, Egypt; basel_post@msn.com; 2 Department of Pharmacology, College of Pharmacy, Najran University, P.O. Box 1988, Najran 55461, Saudi Arabia; 3 Zoology Department, Faculty of Science, Ain Shams University, Abbasseya, Cairo 11566, Egypt; hanaafathy@sci.asu.edu.eg; 4 Clinical Laboratory Science Department, College of Applied Medical Science, Shaqra University, P.O. Box 1383, Al Quwayiyah 11961, Saudi Arabia; aalzamami@su.edu.sa; 5 Cancer Biology and Genetics Laboratory, Centre of Excellence for Advanced Sciences, National Research Centre, Dokki, Cairo 12622, Egypt; cindrella_adel84@yahoo.com; 6 Biochemistry Department, National Research Centre, Dokki, Cairo 12622, Egypt; 7 Department of Pathology, College of Medicine, Taif University, P.O. Box 11099, Taif 21944, Saudi Arabia; basemelesawy2@gmail.com; 8 Cancer Biology Unit, King Fahd Medical Research Center, King Abdulaziz University, P.O. Box 80216, Jeddah 21589, Saudi Arabia; mamostafa@kau.edu.sa; 9 Department of Medical Laboratory Sciences, Faculty of Applied Medical Sciences, King Abdulaziz University, P.O. Box 3646, Jeddah 22252, Saudi Arabia; 10 Department of Molecular Genetics and Enzymology, Human Genetics and Genome Research Institute, National Research Centre, Cairo 12622, Egypt; 11 Botany and Microbiology Department, Faculty of Science, Cairo University, Giza 12613, Egypt; ahmed.ghareeb@med.asu.edu.eg; 12 Department of Clinical Laboratory Sciences, College of Applied Medical Sciences, Taif University, P.O. Box 11099, Taif 21944, Saudi Arabia; a.elaskary@tu.edu.sa; 13 Zoology Department, Faculty of Science, Suez Canal University, Ismailia 41522, Egypt; hebatallah_hassan@science.suez.edu.eg; 14 Department of Pharmaceutical Science, College of Pharmacy, Princess Nourah bint Abdulrahman University, P.O. Box 84428, Riyadh 11671, Saudi Arabia; ngmohamed@pnu.edu.sa (N.G.M.A.); naaltwaijry@pnu.edu.sa (N.A.); 15 Chemistry Department, Faculty of Science, Suez Canal University, Ismailia 41522, Egypt; 16 Institute for Chemistry, Humboldt Universität zu Berlin, Brook-Taylor-Str. 2, 12489 Berlin, Germany

**Keywords:** *Bacillus subtilis*, marine natural product, exopolysaccharide, FTIR, morphological analysis, X-ray diffraction, SEM analysis, AFM analysis, antioxidant, anti-inflammatory, cytotoxicity, acetylcholine esterase activity

## Abstract

In the presented study, *Bacillus subtilis* strain AG4 isolated from marine was identified based on morphological, physiological, phylogenetic characteristics and an examination of 16S rRNA sequences. Novel exopolysaccharide (EPSR4) was extracted and isolated from the *Bacillus subtilis* strain as a major fraction of exopolysaccharide (EPS). The analysis of structural characterization indicated that EPSR4 is a *β*-glycosidic sulphated heteropolysaccharide (48.2%) with a molecular weight (Mw) of 1.48 × 10^4^ g/mole and has no uronic acid. Analysis of monosaccharide content revealed that EPSR4 consists of glucose, rhamnose and arabinose monosaccharide in a molar ratio of 5:1:3, respectively. Morphological analysis revealed that EPSR4 possess a high crystallinity degree with a significant degree of porosity, and its aggregation and conformation in the lipid phase might have a significant impact on the bioactivity of EPSR4. The biological activity of EPSR4 was screened and evaluated by investigating its antioxidant, cytotoxicity, anti-inflammatory, and anti-Alzheimer activities. The antioxidant activity results showed that EPSR4 has 97.6% scavenging activity toward DPPH free radicals at 1500 µg/mL, with an IC_50_ value of 300 µg/mL, and 64.8% at 1500 µg/mL toward hydrogen peroxide free radicals (IC_50_ = 1500 µg/mL, 30 min). Furthermore, EPSR4 exhibited considerable inhibitory activity towards the proliferation of T-24 (bladder carcinoma), A-549 (lung cancer) and HepG-2 (hepatocellular carcinoma) cancer cell lines with IC_50_ of 244 µg/mL, 148 µg/mL and 123 µg/mL, respectively. An evaluation of anti-inflammatory activity revealed that EPSR4 has potent lipoxygenase (LOX) inhibitory activity (IC_50_ of 54.3 µg/mL) and a considerable effect on membrane stabilization (IC_50_ = 112.2 ± 1.2 µg/mL), while it showed cyclooxygenase (COX2) inhibitory activity up to 125 µg/mL. Finally, EPSR4 showed considerable inhibitory activity towards acetylcholine esterase activity. Taken together, this study reveals that *Bacillus subtilis* strain AG4 could be considered as a potential natural source of novel EPS with potent biological activities that would be useful for the healthcare system.

## 1. Introduction

**Figure 1:**
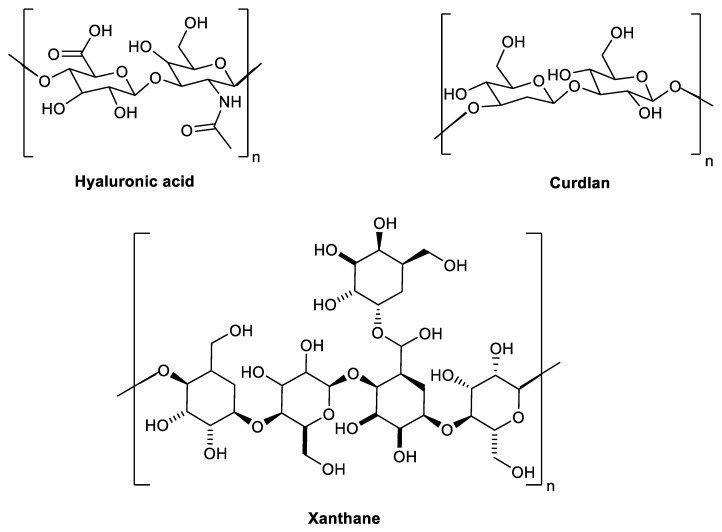
The chemical structure of selected bioactive microbial exopolysaccharides illustrating the diverse in the main chemical scaffold of microbial EPSs.

According to the International Agency for Research on Cancer (IARC) estimations, there were 17.0 million new cancer cases and 9.5 million cancer deaths globally in the last year. The worldwide incidence of cancer is expected to grow to 27.5 million cases and 16.3 million deaths by 2040. Although progress has been made in cancer therapy, reducing its toxicological profile and improving its efficacy, there are still important issues that must be addressed to improve cancer treatment [1]. Indeed, several internationally authorized anti-cancer treatments are natural products or their derivatives that are based on micro-molecules or macro-molecules [2]. Oceans are considered as a gold mine of novel compounds with therapeutic potential, ranging from bacteria to large multicellular creatures [3,4]. One of the most well-known promising compounds are marine carbohydrate-based compounds which have gained the interest of researchers due to their wide range of important pharmacological activities, including antiviral [5,6,7,8], anti-tumor [9,10], antioxidants [11], and anti-inflammatory [12]. Microbial (EPS) are natural soluble or insoluble polysaccharides that are produced in microbial cells and secreted into the extracellular medium, generating a capsule that is enclosed to the surface of the cell or within the fermentation broth [9,10]. EPS can be produced by a variety of microorganisms, including bacteria, cyanobacteria, fungi, and yeasts [13]. Microbial EPSs play a vital biological role in cell survival, including cellular attachment to surfaces, cellular aggregation, cell protection and cellular interactions [14]. Among these bioactive EPSs, cellulose, gellan, dextran, curdlan, levan, alginate, hyaluronic acid, succinoglycan, and xanthan are the most broadly bioactive EPS produced by microorganisms [15]. EPSs are classified according to their functionality into: constructive, redox active, sorptive, surface active, nutritive and informative [16]. EPSs are primarily composed of carbohydrates, such as monomers of D-galactose, L-rhamnose, L-fucose, D-glucose, *N*-acetyl-D-galactosamine, D-glucuronic acid, L-guluronic acid, D-galacturonic acid, D-mannuronic acid, and *N*-acetyl-D-glucosamine (Figure 1) [15]. In addition, EPSs contain noncarbohydrate substituents that give EPSs an anionic nature (such as sulphate, carboxyl, pyruvate and phosphate) and improve the lipophilicity of EPSs and their interactions with cations and other polysaccharides [17,18].

The physicochemical properties of EPSs have attracted attention in many fields, with a particular emphasis on the optimization of manufacturing techniques and bioactivity. The structure of bacteria EPSs is primarily determined by isolates and fermentation requirements [19]. Among different natural polysaccharides, EPSs need less time to produce and a facile extraction technique. Recent research has explored the therapeutic value of EPSs as well as their pharmaceutical applications [19]. EPSs have acquired a myriad of efficient applications in skin care regimes, as dietary supplements, and in pharmaceutics. In the pharmaceutical industry, bacterial EPSs have been examined as nanopatterned scaffolds for their activity to stimulate differentiation of embryonic stem cells and cellular proliferation [20].

To date, there are more than 17 marine-based approved drugs on the market worldwide, including six marine carbohydrate-based drugs [15]. Marine EPS sourced from bacteria have also shown potential in cell therapy and tissue engineering. When compared to other polysaccharides from eukaryotes, marine EPSs can be developed in bioreactors under completely controlled conditions [3]. Marine EPSs have been recently noted for their exceptional anti-tumor, antioxidant and immune-modulatory activities, attracting attention as a favorable source of potential therapeutic therapy [21]. EPSs are derived from bacteria from extreme marine habitats and therefore possess exclusive properties, due to their ability to adapt to extreme conditions [22]. Recently, G. liu and his colleagues reported the activity of a novel marine bacterial EPS (EPS11) on hepatic cancer invasion and metastasis. The results showed that EPS11 has considerable inhibitory activity toward cell invasion, migration, and adhesion of hepatic cancer cells. This activity was attributed to the ability of EPS11 to downregulate the proteins involved in the extracellular matrix–receptor interaction pathway and to modulate collagen I activity [15]. Another study reported that EPS from *Lactobacillus gasseri* (EPS G10) inhibits cells division and proliferation. Indeed, EPS G10 at 400 μg/mL exhibited superior cytotoxicity activity and induced apoptosis by elevating the expression of BAX apoptotic factor and caspase 3 [23]. EPS-M41, isolated from marine *Pediococcus pentosaceus*, showed considerable cytotoxicity efficacy toward Caco-2 and MCF-7 cancer cell lines [24]. The EPS extracted from *Lactobacillus plantarum* NCU116 showed the ability to induce apoptosis in intestinal cancer cells by elevating the expression of proapoptotic genes, including c-Jun, Fas, and FasL, by targeting the Toll-like receptor-2 (TLR-2) [25]. Azad et al. have explored the anti-proliferative function of EPS extracted from mutant *Lactobacillus delbrueckii* in tumor cells. The results revealed that the extracted EPS downregulated the carcino-embryonic antigen (CEA) levels in a tumor-induced rat model [26]. The EPS extracted and purified from *Lactobacillus pentosus* LZ-R-17 showed remarkable immunomodulatory activity via boosting the capability of macrophage cells, increasing phagocytosis and stimulating the secretion of proinflammatory cytokines [27]. Lin et al. investigated the antioxidant activity of EPS (DeinoPol) isolated from *Deinococcus radiodurans* R1 and demonstrated DeinoPol as the first deinococcal EPS that might be safely used in cosmetics and pharmaceuticals [28]. The inhibitory activity of EPSs from *Bifidobacterium bifidum* WBIN03 (B-EPS) toward lipid peroxidation of was investigated by Li et al. [29]. The results revealed that B-EPS possesses a considerable inhibitory effect (13.48 ± 1.74%) compared to ascorbic acid at the same concentration (23.20 ± 1.41%) [29]. The anti-Inflammatory activity of EPS fractions (GR-1 to GR-21) produced from the polluted soil bacteria was recently explored by Gangalla and his colleagues. The results indicated that EPSs GR-2, GR-5, and GR-1 (65 ± 0.14, 61 ± 0.15 58 ± 0.38, respectively) possess potent anti-inflammatory activity compared to indomethacin drug [30]. Furthermore, a recent study showed that administration of EPS extracted from *B. subtilis* suppresses the T-cell activation and control T cell-mediated immune responses in several inflammatory diseases [31]. Furthermore, Asker et al. showed that the EPS fraction extracted from *Achromobacter piechaudii* NRC2 bacteria possesses potent and selective anti-cyclooxygenase COX-1&2 activity, together with considerable antioxidant properties [7].

Based on the abovementioned facts, our ongoing research aimed at discovering and isolating novel EPS from marine sediment. The structural characterization of isolated EPS has been investigated. Furthermore, the activity of the isolated EPS (EPSR4) was explored in vitro and screened toward antioxidant, anti-inflammatory, cytotoxicity, and anti-Alzheimer activities.

## 2. Results

### 2.1. Identification of the Bacterial Isolates

**Figure 2:**
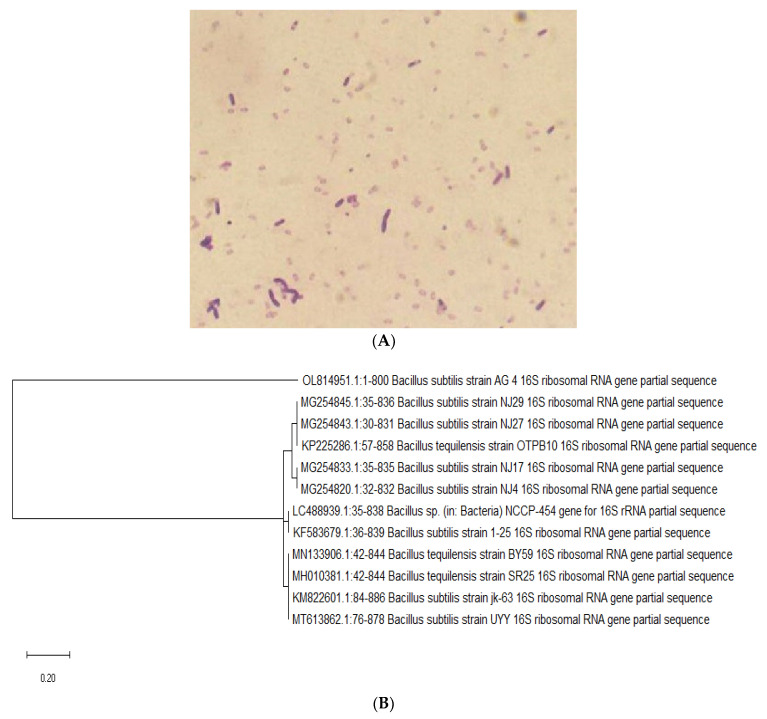
Identification of the bacterial strain that produce EPS. (**A**) Gram positive stain *Bacillus subtilis* strain AG4. (**B**) Phylogenetic tree of the partial sequence of 16S rRNA of the local isolate respects to closely related sequences available in Gen Bank databases.

In the present study, the bacterial samples were obtained from marine sediment in the Red Sea and were grown in a media containing glucose, CaCO_3_, NH_4_NO_3_, KH_2_PO_4_, K_2_HPO_4_, hydrated MgSO_4_, hydrated MnSO_4_., and yeast extract [32]. Based on their important colony morphological and biochemical features, seven bacterial isolates were selected and screened for their ability to produce EPS_S_ with high antioxidant activity [33]. Among these isolates, three strains were found to possess the ability to produce EPSs in a satisfactory amount. The highest EPS yield was obtained from the marine bacterium isolated from (R4) (8.12 g/L). Based on these results, we have focused our investigations on the R4 bacterium isolate. Standard biochemical, physiological, and morphological investigations showed that the R4 bacterium strain is a Gram-positive Bacilli with an irregular large colony, a dull surface and smooth texture and has no pigments (Figure 2A). The R4 bacterium isolate showed positive results in the catalase test, Voges–Proskauer test, Simon citrate test, and Nitrate reduction. The identification of the bacterial isolate was confirmed by phylogenic analysis using PCR analysis, utilizing the 16S rRNA 5′-TCCGTAGGTGAACTTTGCGG-3′ primer [34,35] (Figure 2B). The nucleotide sequence results were analyzed and compared to the GenBank database utilizing the BLAST program. The phylogenetic tree was generated by aligning sequences with the greatest similarity to the rRNA sequences of the isolated bacterial and the obtained sequences of the rRNA gene was identified as the *Bacillus subtilis* strain AG4 and deposited in the nucleotide sequence database (https://www.ncbi.nlm.nih.gov/ accessed on 15 December 2021) with the accession number OL814951 (DDBJ/EMBL/GenBank).

### 2.2. Production and Purification of EPSR4

The R4 bacterium isolate was used to produce the exopolysaccharide EPSR4 in a yield of 8.12 g/L. The obtained crude product was engaged to a purification step through fractionation and precipitation processes. Thus, the crude EPSR4 was dissolved in deionized water and the obtained solution was subsequently dialyzed for 72 h against deionized water. After the dialyzed solution was concentrated with ultrafiltration membrane of 100 KD, EPSR4 was fractionated and precipitated by four-fold volume of absolute ethanol (pre-cooled) to afford a faint gray solid of EPSR4 in 83.4% yield. The obtained EPSR4 product was soluble in water, but not soluble in absolute ethanol. The EPSR4 concentration was evaluated using the phenol-sulfuric acid protocol at A490 [36].

### 2.3. Molecular Weight and Chemical Composition of EPSR4

**Figure 3:**
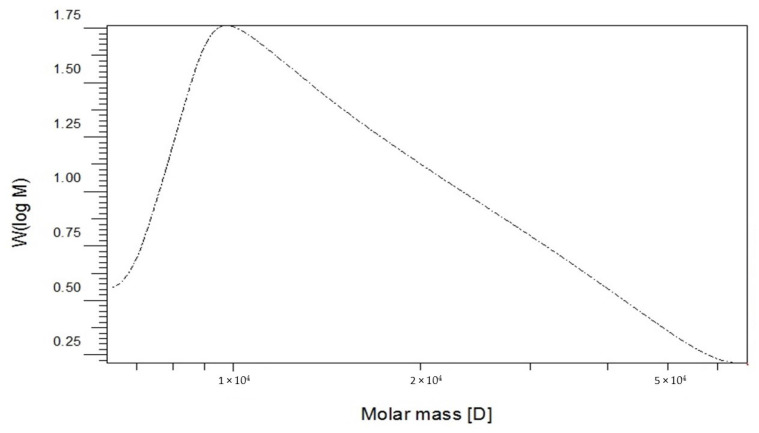
Gel permeation chromatography analysis of the semi-purified EPSR4 from R4 bacterium isolate.

We have deeply investigated the chemical composition and the structural characterization of EPSR4. Our analysis revealed that the obtained EPSR4 is sulfated polysaccharide (48%) and has no glucuronic acid in the structure. The gel permeation chromatographic (GPC) analysis showed that EPSR4 has an average molecular weight of 1.48 × 10^4^ g/mole, while it has number average molecular weight (Mn) of 1.3 × 10^4^ g/mole (Figure 3). Furthermore, the GPC chromatogram indicated that EPSR4 is an extensively dispersed molecule with a polydispersity index (PI) of 1.3. Analysis of the carbohydrate showed that EPSR4 belongs to the heteropolysaccharide EPS family. Analysis of the monosaccharaide composition demonstrated that EPSR4 is composed of glucose, rhamnose, and arabinose in a molar ratio of 5:1:3, respectively (Figure S1). These results indicate that EPSR4 has an acidic nature, which could be attributed to the heteropolysaccharide and/or the high levels of sulfation.

### 2.4. Structural Characterization of EPSR4 by FT-IR and UV-Vis Analysis

**Figure 4:**
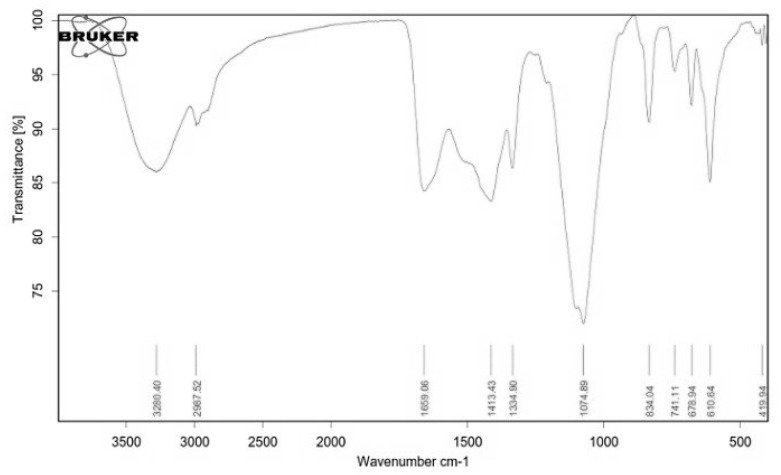
The Fourier transform infrared spectrum (FTIR) of the EPSR4.

We further explored the FTIR spectrum of EPSR4 which indicated many peaks from 610 to 3280 cm^−1^ (Figure 4). The spectrum showed a broad band at 3280 cm^−1^ which corresponded to the vibration of the hydroxyl groups in the sugar residues [37]. The band at 2987 cm^−1^ was attributed to the symmetrical and asymmetrical stretching vibration of the C–H in the sugar residues [38]. The absorption peak at 1659 cm^−1^ was severed to the stretching of the carbonyl (C=O) group [39]. The band at 1413 cm^−1^ represented CH_2_ in the sugar moiety, and the absorption at 1334 cm^−1^ was attributed to the carboxylate groups [40]. The peak at 1074 cm^−1^ indicated the SO_3_ group. Moreover, the band at 834.04 cm^−1^ indicated a glucosyl residue with *β*-pyranose form [41]. The absence of peaks at 918 and 843 cm^−1^ indicated that EPSR4 has no *α*-glycosidic linkages [42,43]. Therefore, the infrared spectrometry analysis indicated that EPSR4 is more likely to belong to a *β*-glycosidic heteropolysaccharide [44]. The UV analysis of EPSR4 did not show a peak at 260 nm, indicating the absence of proteins after the purification process (Figure S2).

### 2.5. Morphological Analysis of EPSR4

**Figure 5:**
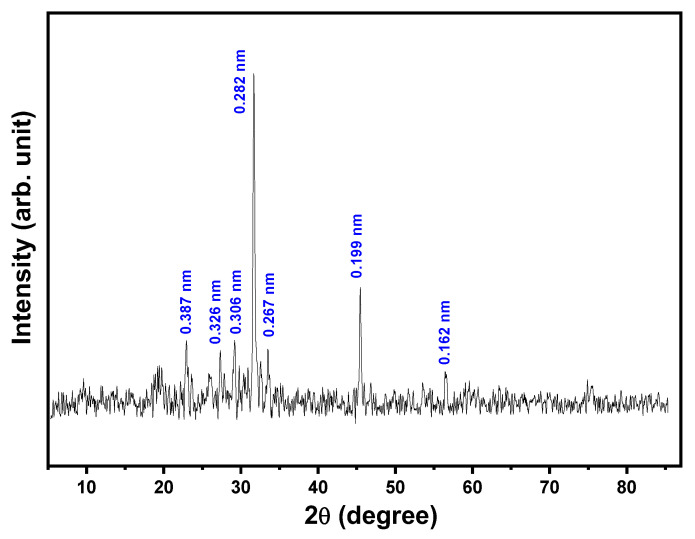
X-ray diffraction pattern of EPSR4.

The crystalline structure of the EPSR4 has been evaluated utilizing the recorded XRD pattern. As shown in Figure 5, the XRD diffractogram revealed that the EPSR4 possesses intense diffraction peaks at different positions, giving rise to a polycrystalline nature of high crystallinity degree (87.7%) and a very low amorphous contribution of 12.3% in the extracted EPS sample. The highest diffraction peaks were detected at positions 2*θ* (22.948, 27.34, 29.180, 31.676, 33.495, 45.471, and 56.53°), which correspond to inter-planar distance *d* (0.387, 0.326, 0.306, 0.282, 0.267, 0.199, and 0.162 nm). The average size of the nanostructure EPSR4 crystallites has been calculated using the Scherrer equation and found to be (~39.59 ± 4 nm), which is lower than that recorded previously in the literature [45,46]. It is worth mentioning that the obtained high crystallinity of EPSR4 compared to exopolysaccharides extracted from other resources could be attributed to the high molecular weight of EPSR4 [42,46]. Furthermore, the high degree of crystallinity could be associated with the declared anti-inflammatory and anticancer activity of the EPSR4 [47,48]. The high crystallinity of EPSR4 enhances the interaction between the cancer nucleus and EPSR4 molecules besides affecting the permeability via the cell membrane [48].

**Figure 6:**
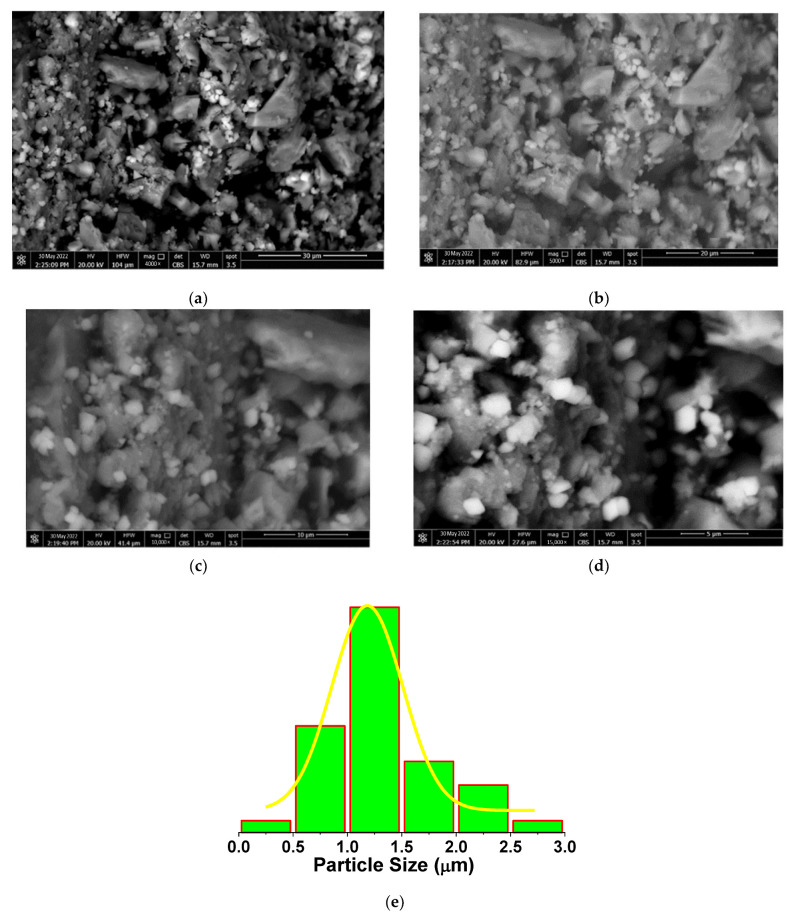
SEM micrographs of the EPSR4 at different magnifications, ×4000 (**a**), ×5000 (**b**), ×10,000 (**c**), ×15,000 (**d**), and the particle size distribution (**e**).

To further explore the morphological features of the EPSR4, we performed a scanning electron microscopy (SEM) analysis. The recorded SEM micrographs of different magnification scales are shown in Figure 6. The SEM analysis revealed the formation of a macro-crystallite structure by the agglomeration of densely packed nanostructured polysaccharide particles by hydrogen bonding, owing to the high molecular weight. Additionally, as observed from the SEM micrographs, these macro-crystallites are intervened by a significant degree of porosity of about ~12.68%.

**Figure 7:**
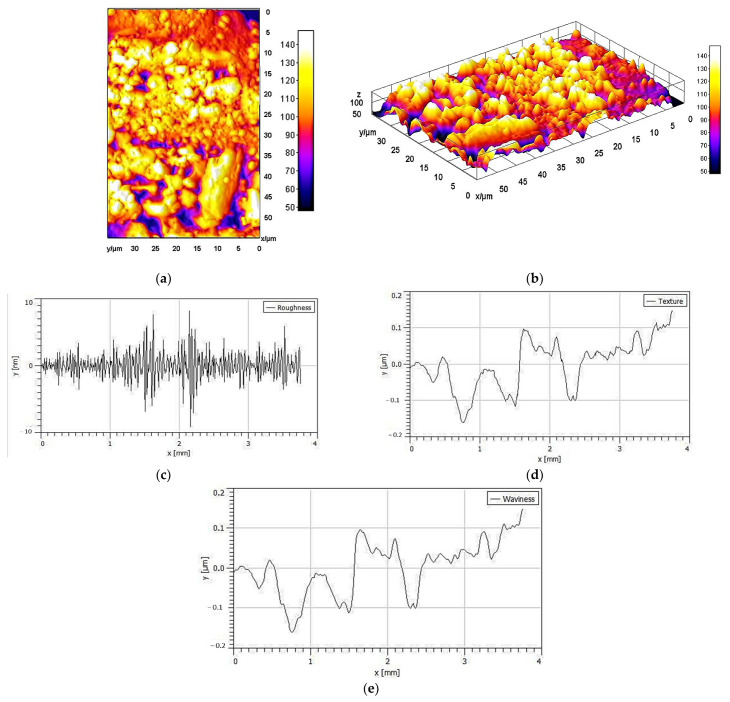
Atomic force microscopic analysis of EPSR4 representing the topology in 2D (**a**) and 3D (**b**), roughness (**c**), texture (**d**), and waviness profile (**e**) of EPSR4.

For further valuable inspections of the nanoscale morphology of the EPSR4 in the liquid state, atomic force microscopic (AFM) analysis has been employed to pick up the high-resolution images of the EPSR4 surface. As indicated in Figure 7, AFM analysis revealed a highly textured, wavy, and rough surface morphology and topology, indicating the cross-linking and clustering of the polysaccharide chains. The estimated maximum height was about 29.16 nm, which is higher than the length of a single EPS chain (0.1–1 nm), indicating that EPSR4 molecular chains intend to create polymer clusters [49]. Taken together, the morphological analyses indicate that ESPR4 has a high degree of crystallinity with a significant degree of porosity, and its aggregation and conformation in the lipid phase might have a significant impact on the bioactivity of EPSR4.

### 2.6. Antioxidant Activity

**Figure 8:**
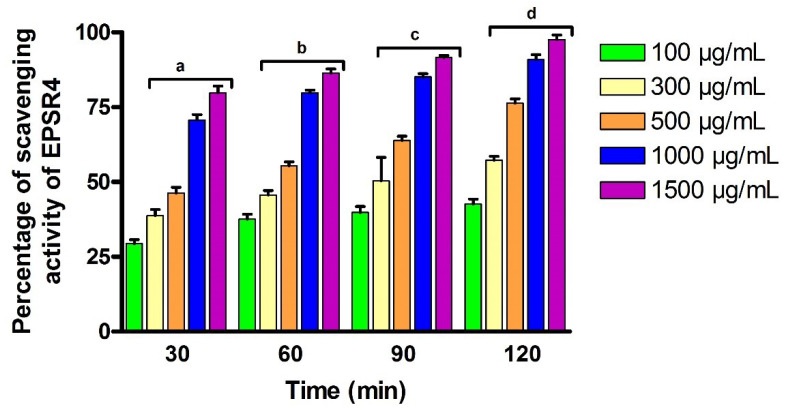
The activity of EPSR4 to scavenge the free radical of DPPH at different time intervals. Data are presented as mean ± SD. ^a^ *p* < 0.05 versus concentration of 100 μg/mL in 30 min, ^b^ *p* < 0.05 versus concentration of 100 μg/mL in 60 min, ^c^ *p* < 0.05 versus concentration of 100 μg/mL in 90 min, ^d^ *p* < 0.05 versus concentration of 100 μg/mL in 120 min.

Natural polysaccharides have been considered as a potential source of antioxidants. With the goal to investigate the antioxidant activity of EPSR4, the DPPH assay was performed to investigate the ability of EPSR4 at different concentrations (100, 300, 500, 1000, 1500 µg/mL) to scavenge the DPPH free radicals at a wide range of time intervals (30, 60, 90 and 120 min). Toward this aim, we have followed a methodology which was previously reported by Gülcin et al. [50]. Thus, the EPSR4 at different concentrations was treated with a solution of DPPH and the bleaching absorption of DPPH free radical was assessed after incubation, in the dark for 30, 60, 90 and 120 min, by spectrophotometer at a wavelength of 517 nm. As shown in Figure 8, the results revealed that the entire antioxidant activity of EPSR4 was increased in a dose-(100, 300, 500, 1000 and 1500 µg/mL) and time-dependent manner. At concentrations of 100, 300 and 500 µg/mL, the scavenging activity of EPSR4 to bleach the DPPH free radical was 29–46% after 30 min, and this ratio was increased over time by ~45% to reach 42–76% at 120 min. However, at 1000 and 1500 µg/mL concentrations, the scavenging activity of EPSR4 was 70–80% after 30 min, and this ratio was enhanced over exposure time to DPPH by ~28% to reach 90–98% after 120 min. Our results demonstrated that the highest antioxidant activity obtained was at 1500 µg/mL after 120 min (98%) (*p* < 0.05). Based on these results, we have assessed the IC_50_ value (half maximal inhibitory concentration), the concentration of the sample that scavenge 50% of the DPPH free radical, of EPSR4 and was found to be around 300 µg/mL.

**Figure 9:**
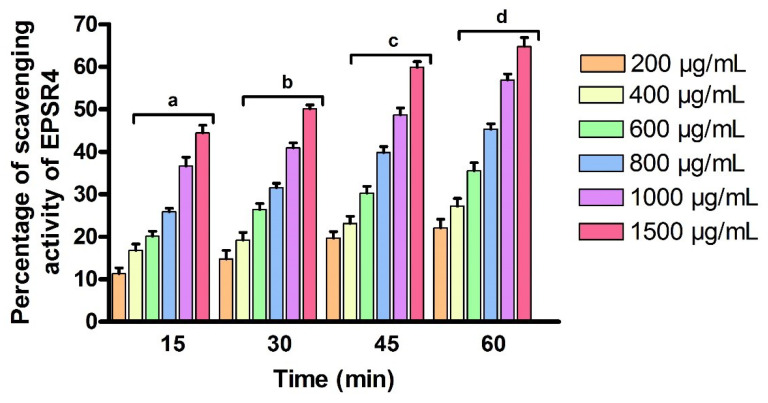
The activity of EPSR4 to scavenge the free radical of hydrogen peroxide at different time intervals. Data are presented as mean ± SD. ^a^ *p* < 0.05 versus concentration of 200 μg/mL in 15 min, ^b^ *p* < 0.05 versus concentration of 200 μg/mL in 30 min, ^c^ *p* < 0.05 versus concentration of 200 μg/mL in 45 min, ^d^ *p* < 0.05 versus concentration of 200 μg/mL in 60 min.

Next, we have evaluated the antioxidant activity of EPSR4 by investigating its ability to remove the hydrogen peroxide free radical. One important feature of antioxidants is the ability to remove free radicals by donating a hydrogen atom to the free radical and altering them to a non-reactive species. To this end, EPSR4 was analyzed for its ability to scavenge the free radicals, employing the hydrogen peroxide assay as previously reported. The activity of EPSR4 was examined at different concentrations (200, 400, 600, 800, 1000, 1500 µg/mL) over different time intervals (15, 30, 45 and 60 min). In this regard, EPSR4 at different concentrations was treated with a solution of hydrogen peroxide and the absorption of the resulting solution was assessed by a spectrophotometer at a wavelength of 230 nm after incubation in dark over different time intervals. As illustrated in Figure 9, our findings revealed that the scavenging activity of EPSR4 toward the hydrogen peroxide free radicals was time- and dose-dependently increased. At concentrations of 200, 400, 600, and 800 µg/mL, the ability of EPSR4 to scavenge the free radical of hydrogen peroxide was increased by ~47% over time to reach 22–45% after 60 min. On the other hand, at 1000 and 1500 µg/mL, the scavenging activities of EPSR4 was increased over time exposure by ~30% to reach 57–65% after 60 min. We observed that the highest antioxidant activity was obtained at 1500 µg/mL after 60 min (65%) (*p* < 0.05), and the IC_50_ value was defined to be 1500 µg/mL after 30 min.

### 2.7. Cytotoxic Activity against Different Cancer Cell Lines

**Figure 10:**
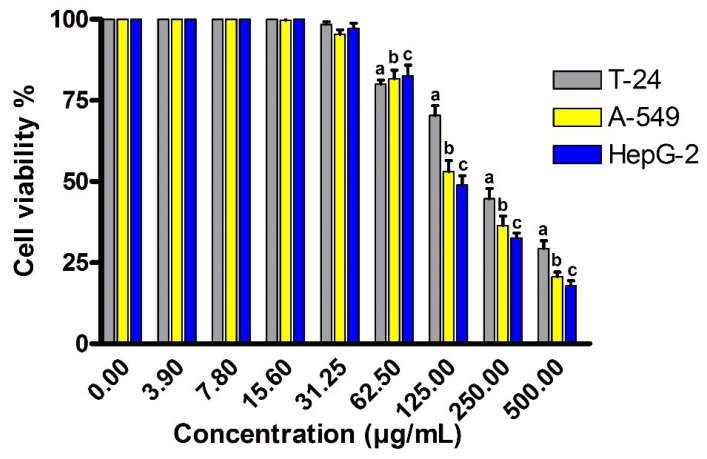
Evaluation of the cytotoxic activity of EPSR4 against different mammalian cancer cell lines; bladder carcinoma T-24, lung cancer A-549 and hepatocellular carcinoma (Hep-G2). Data are presented as mean ± SD. ^a^ *p* < 0.05 versus concentration of 0 μg/mL in bladder carcinoma T-24, ^b^ *p* < 0.05 versus concentration of 0 μg/mL in lung cancer A-549, ^c^ *p* < 0.05 versus concentration of 0 μg/mL in hepatocellular carcinoma (Hep-G2).

Natural polysaccharides are considered as potent antitumor agents with high bioactivity and chemoprevention. Contrasted to traditional antitumor therapy, natural polysaccharides are characterized by the immuno-regulation function and low toxicity. Several polysaccharides have been recognized as safe and non-toxic anticancer agents. Recently, the potency of natural exopolysaccharide as antitumor has been irrefutably demonstrated [51]. Based on these facts, we have screened the cytotoxicity effect of EPSR4 fraction against the proliferation of transitional cell carcinoma of the urinary bladder T-24, adenocarcinomic lung tissue A-549 and human hepatoma Hep-G2 cell line. Toward this, the cells were treated with different concentrations of EPSR4 and incubated for 24 h. As presented in Figure 10, EPSR4 treatment demonstrated considerable cytotoxicity toward the cell growth of all investigated cancer cell lines (*p* < 0.05) at concentrations more than 62.5 µg/mL. Toward the mammalian urinary bladder T-24 cancer cell line, EPSR4 showed the lowest cytotoxic effect, with an IC_50_ value of 244 ± 6.9 µg/mL as compared to cisplatin IC_50_ = 31 ± 1.8 µg/mL. On the other hand, EPSR4 exhibited potent cytotoxic effect on both lung cancer A-549 and hepatocellular carcinoma Hep-G2, with IC_50_ value of 148 ± 5.8 µg/mL and 123 ± 4.3 µg/mL, respectively, compared to cisplatin (IC_50_ of 4.08 ± 0.46 µg/mL for A-549 and 1.29 ± 0.17 µg/mL for Hep-G2). These results indicate that EPSR4 fraction exerts significant cytotoxic effects via suppression of tumor growth in lung cancer and human hepatoma cell lines. Nevertheless, understanding the mode of action and signaling mechanism of EPSR4 against hepatic carcinoma and lung cancer is important for designing novel and effective treatments.

### 2.8. Anti-Inflammatory Activity

**Figure 11:**
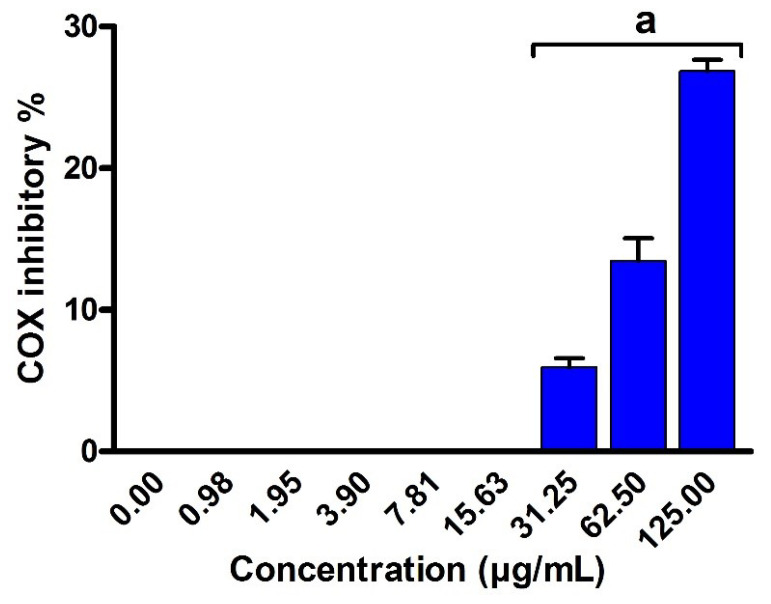
Assessment of the inhibitory activity of EPSR4 against COX-2 enzymatic activity. Data are presented as mean ± SD. ^a^ *p* < 0.05 versus concentration of 0 μg/mL.

The inflammation process is initiated as a protective defense in response to various intrinsic and extrinsic stimuli. Persistent inflammation has been associated with various diseases including neurological disorders, cancer, and cardiovascular diseases. Among these regulators of arachidonic acid metabolism, the over expression of 5-LOX and COX-2 has been linked to various inflammatory diseases [52]. Targeting of 5-LOX/COX-2 enzymes have been emphasized based on their ability to reduce the production of leukotrienes and prostaglandin [53]. COX-2/5-LOX inhibition is based on the prevention of formation of both prostaglandin and leukotrienes. COX-2 inhibitors exhibit anti-inflammatory effect by preventing the conversion of arachidonic acid to prostaglandin. Arachidonate 5-lipoxygenase inhibitors prevent the action of the arachidonate 5-lipoxygenase (5-LOX) enzyme, that is in charge for the generating the inflammatory leukotrienes. Based on these facts, we were interested to investigate the anti-inflammatory activity of EPSR4 by evaluating its inhibitory potency toward COX-2 and 5-LOX enzymes. To assess the anti-inflammatory role of EPSR4, we have used ibuprofen and celecoxib as control anti-inflammatory drugs. Ibuprofen is hydroxylamine and hydroxamic acid derivatives with known non-steroidal anti-inflammatory activity displayed inhibitory activity both in vitro and in vivo against COX-2/5-LOX [52]. While celecoxib is a non-steroidal anti-inflammatory drugs with selective COX-2 inhibitory activity which is the key enzyme for the synthesis of prostaglandins from arachidonic acid [54]. The inhibitory activity of EPSR4 toward COX-2 enzyme was assessed following the previously reported methods by Petrovic and Murray and Amessis-Ouchemoukh et al. [55,56]. As shown in Figure 11, EPSR4 exhibited a moderate inhibitory activity toward COX-2 enzyme in a dose-dependent manner (*p* < 0.05) with IC_50_ > 125 μg/mL, while celecoxib showed IC_50_ of 0.28 μg/mL.

**Figure 12:**
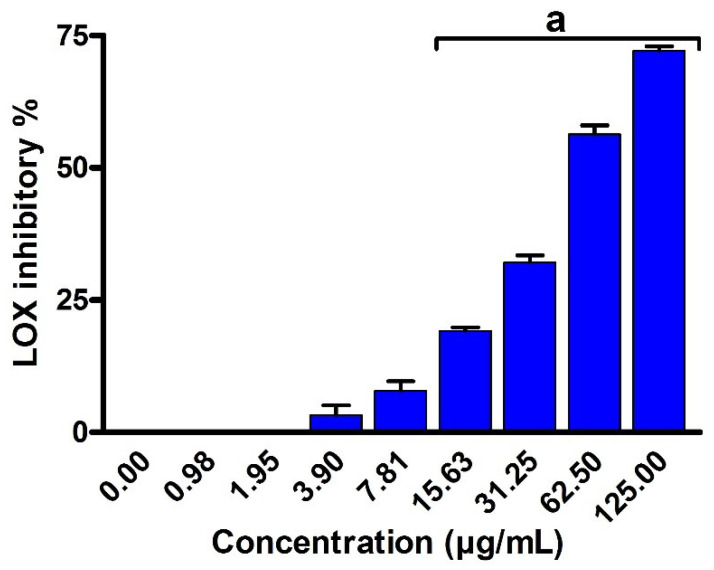
Evaluation of the inhibitory activity of EPSR4 against 5-LOX enzymatic activity. Data are presented as mean ± SD. ^a^ *p* < 0.05 versus concentration of 0 μg/mL.

Next, we have examined the inhibitory activity of EPSR4 toward 5-LOX enzyme following the reported method by Granica et al. [57]. The results demonstrated that EPSR4 extract has a potent inhibitory activity toward 5-LOX enzyme (*p* < 0.05) with IC_50_ of 54.3 ± 2.1 μg/mL, compared to ibuprofen with IC_50_ of 1.5 ± 1.3 μg/mL (Figure 12). Taken together, these results indicate that EPSR4 has a potent anti-inflammatory activity which could be attributed to its high inhibitory activity toward 5-LOX enzyme.

**Figure 13:**
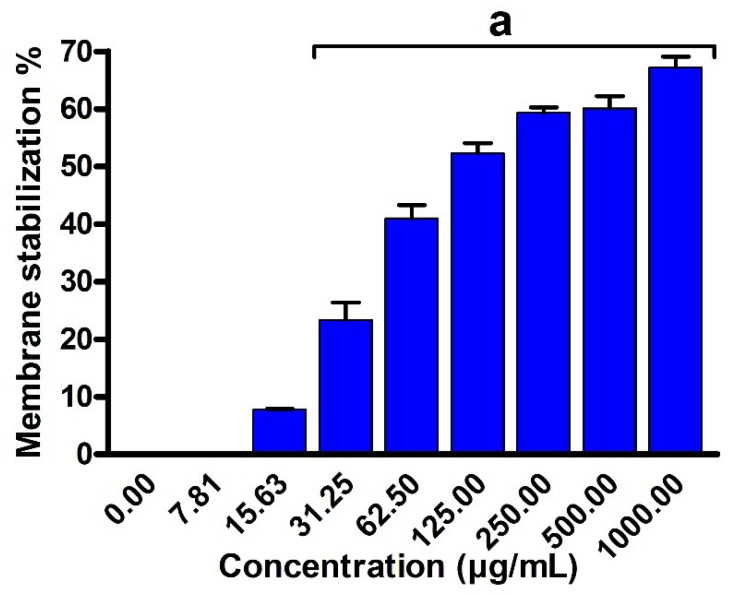
Assessment of the inhibitory activity of EPSR4 against hemolysis activity (membrane stability). Data are presented as mean ± SD. ^a^ *p* < 0.05 versus concentration of 0 μg/mL.

In order to affirm our results, we have investigated the effect of EPSR4 extract on the membrane stability or hemolysis activity. Membrane stabilization is also a well-documented cause of inflammation. To evaluate the inhibitory activity of EPSR4 toward hemolysis activity, we followed the reported method by Shinde et al., which is based on erythrocyte hemolysis induced by hypotonic solution [58]. In our experiment, we utilized indomethacin as a standard control drug. Indomethacin, a nonsteroidal anti-inflammatory drug, is a powerful prostaglandin synthesis inhibitor which reduces prostaglandin production by inhibiting the activity of COX1&2. As indicated in Figure 13, EPSR4 showed a potent inhibitory activity toward the hemolysis activity or membrane stabilization (*p* < 0.05) with IC_50_ of 112.2 ± 1.2 µg/mL, compared to indomethacin with IC_50_ of 17.02 ± 0.82 µg /mL. These results affirm the anti-inflammatory effect of EPSR4.

### 2.9. Acetylcholinesterase (AChE) Inhibition

**Figure 14:**
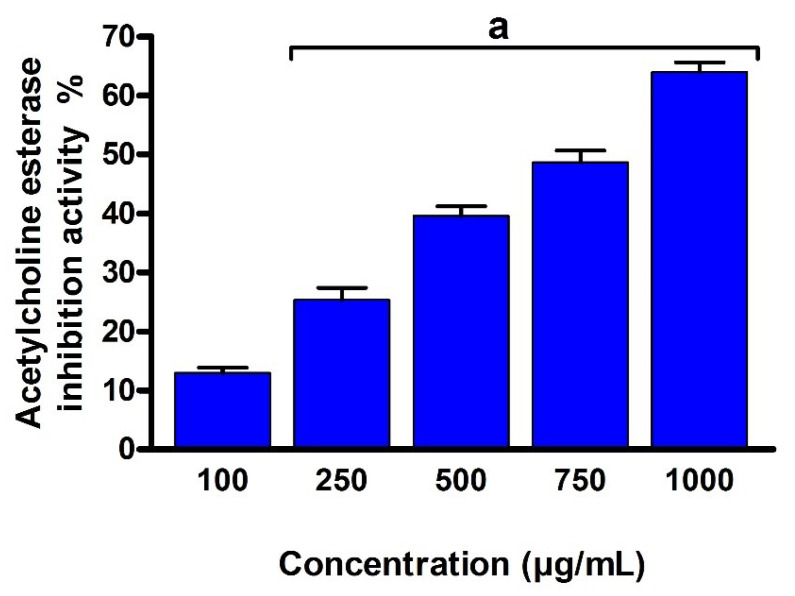
Evaluation of the inhibitory activity of EPSR4 against acetylcholinesterase activity. Data are presented as mean ± SD. ^a^ *p* < 0.05 versus concentration of 100 μg/mL.

The pathogenesis of Alzheimer’s disease has been linked to a deficiency in the brain neurotransmitter acetylcholine, which is based on cholinergic system abnormalities with intellectual impairment [59]. In order to further explore the biological potency of EPSR4, we investigated the inhibitory potency of EPSR4 toward acetylcholine esterase activity. To assess the activity of EPSR4, we followed the reported assay by Monserrant and Bianchini. Thus, acetylcholinesterase enzyme was treated with EPSR4 at different concentrations and the inhibitory activity was assessed by a spectrophotometer. In our assay, we used Eserine as a standard control inhibitor for acetylcholinesterase enzyme. As illustrated in Figure 14, EPSR4 showed a dose-dependent and moderate inhibitory activity toward acetylcholinesterase activity (*p* < 0.05) with an IC_50_ of 786.38 µg/mL, compared to Eserine with IC_50_ value of 0.09 µg/mL. Considering previously reported EPSs, these results indicate that EPSR4 could be considered as a promising modulator for acetylcholine esterase activity. Further studies should be performed to investigate the bioavailability and biocompatibility of EPSR4 in an in vivo animal disease model.

## 3. Discussion

Marine bacteria are considered as a gold mine of novel natural compounds with therapeutic potential. Accordingly, several studies have been conducted to extract and explore the activity of their bioactive natural products including polysaccharides. Polysaccharides are high molecular weight organic compounds that have certain significant chemical and physical properties which facilitate their functional properties, including production capability, flocculation gelling, thickening, film formation, and emulsion stabilization [17,18]. Among various microbial polysaccharides, recent studies have focused on discovering and exploring microbial EPs with novel biological activities. EPSs are hydrogenated polysaccharides that contain DNA and proteins with important properties, including thickening, gelling, and biocompatibility. Such unique functional properties facilitate their industrial and therapeutic application [17,18]. The conditions of bacterial fermentation determine the final chemical composition (molecular weight, conformation, glycosidic linkage, monosaccharide composition, etc.) of EPSs, which defines the biological potency of the biosynthesized EPS [60,61].

**Figure 15:**
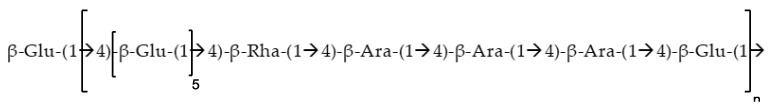
The predicted structure of EPSR4.

EPSs isolated from marine bacterial strains have been reported to possess valuable therapeutic bio-functionality, including cytotoxicity, anti-inflammatory, antioxidants, and immunomodulatory agents [23]. Based on these facts, our ongoing research aimed at isolating novel EPS from marine sediment and exploring its biological activities. In our study, seven marine-derived bacterial isolates from the Red Sea were isolated and selected for a screening program to produce EPSs, based on their unique morphological characteristics and their ability to produce EPSs with highest antioxidant activity [33]. Accordingly, three isolates were discovered to possess the ability to produce EPSs in a reasonable amount. Among these isolates, the marine bacterium isolated from (R4) showed the ability to produce EPSs with the highest yield (8.12 g/L). Investigations on the bacterium isolate R4 revealed that the R4 isolate is *Bacillus subtilis* strain AG4. These results were based on biochemical, physiological, morphological, and phylogenic analysis [34,35]. We have focused first on the structural characterization of the isolated EPSR4. The GPC analysis showed that EPSR4 has a *Mw* of 1.48 × 10^4^ g/mole and number average molecular weight of 1.12 × 10^4^ g/mole. These results are in line with Xia et al., who reported that the EPSs have Mw varying from 10 to 6000 kDa [62]. Analysis of monosaccharide showed that EPSR4 is a heteropolysaccharide which consists of glucose, rhamnose, and arabinose. Furthermore, FT-IR spectra revealed that EPSR4 belongs to *β*-glycosidic polysaccharide. Exploration of the morphological characteristics (XRD, SEM, AFM) of EPSR4 revealed that the isolated polysaccharide has a high degree of crystallinity with a significant degree of porosity, and its aggregation and conformation in the lipid phase might have a significant impact on the bioactivity of EPSR4. Based on these structural characterization analyses, the structure of EPSR4 could be predicted as shown in Figure 15. Further analysis should be performed including 1D and 2D NMR analysis and methylation analysis in order to obtain more insights into the structure of EPSR4. To date, no studies have been found that have been able to determine the exact structure of extracted EPS. Mostly, the reported structures are mainly estimated but not confirmed [49,63,64,65,66]. This could be attributed to the high average molecular weight of EPS (>1 × 103 Da). This would require a highly technical and experimental analysis and chemistry [67].

Next, we explored and screened the biological potency of EPSR4 by evaluating its antioxidant, cytotoxicity, anti-inflammatory and anti-Alzheimer activities. Assessment of the antioxidant activity indicated that EPSR4 possesses 97.6 ± 1.5% after 120 min toward scavenging of DPPH free radicals at 1500 µg/mL concentration, while it showed 64.8 ± 2.1% scavenging activity toward hydrogen peroxide free radicals at 1500 µg/mL concentration after 30 min (Figure 8 and Figure 9). The antioxidant activity was examined in comparison to ascorbic acid as a reference agent (Figure S3). Previous studies showed that the partly purified EPSs of *Lactobacillus plantarum* YML009 possess the ability to scavenge ROS and upgrade enzymatic and non-enzymatic antioxidant activities [68]. Furthermore, the EPSs isolated from *Lactobacillus delbrueckii* sp. *bulgaricus* SRFM-1 demonstrated considerable antioxidant activity [65]. Several reports attributed the scavenging activity of EPS toward free radicals to its ability to bind to the radical, and, thus, terminating the radical chain cascade reaction [69]. The EPS has the ability to chelate metal ion, such as Fe^2+^ and Cu^2+^, and inactivate these ions allowing the hydroxyl production to begin. Moreover, owing to the elevated charge density of the carbon atoms in the heterocyclic ring, the sulfate group could improve the scavenging activity of the radicals [51]. Our results revealed that EPSR4 possesses a high content of sulphate (about 48%), which could be associated with the observed antioxidant activity. Our findings are in accordance with Mohamed et al., who explored the antioxidant activity EPS isolated from *Bacillus* sp. NRC5. This study indicated that the extracted EPS has considerable scavenging activity toward Fe^2+^ ion, nitric oxide radical, and lipid peroxidation. The authors have attributed this activity to the unique chemical composition of this EPS [70]. To this end, our analysis revealed that EPS consists of glucose, rhamnose, and arabinose. The aldehyde groups of these monosaccharides act as reducing agent, which could contribute to the antioxidant ability of EPSR4 [64]. Notably, the observed antioxidant activity could also be attributed to the ability of EPSR4 to up-regulate the expression of superoxide dismutase and support the formation of glutathione [71].

Furthermore, we have evaluated the inhibitory activity of EPSR4 on the proliferation of three types of cancer cell lines, namely urinary bladder T-24, adenocarcinomic lung tissue A-549 and human hepatoma Hep-G2 cell (Figure 10). Our results demonstrated that EPSR4 has IC_50_ of 244 ± 6.9 µg/mL towards the T-24 cell line, while the IC_50_ toward A-549 cell line has 148 ± 5.8 µg/mL and for HepG-2 cell line, 123 ± 4.3 µg/mL, as compared to cisplatin (IC_50_ of 31 ± 1.8 µg/mL, 4.08 ± 0.46 µg/mL, 1.29 ± 0.17 µg/mL for T-24, A-549 and Hep-G2, respectively). These results reveal that EPSR4 has substantial cytotoxic activity toward both lung cancer and human hepatoma cell lines. Several studies have demonstrated the antitumor activity of microbial EPS. Sungur et al. recently showed that the EPS isolated from *Lactobacillus gasseri* G10 at 400 μg/mL significantly inhibits the proliferation of HeLa cells by elevating the expression of both and caspase 3 proteins, which induce apoptosis [72]. In addition, the EPS extracted from *Pediococcus pentosaceus* M41 demonstrated antiproliferative activity toward both colon Caco-2 cell line (77.5% tumor inhibition) and breast cancer cells (MCF-7) (46.4% antitumor inhibition) [24]. Interestingly, Zhou et al. found that the EPS isolated from *Lactobacillus plantarum* NCU116 up-regulates the expression of several pro-apoptotic genes in rat intestinal epithelial cancer cells [25]. Several reports indicated that the cytotoxicity activity of EPS could be associated with the high molecular weight of EPSs, which facilitates their interactions with the receptors of cancer cells and, thus, modulation signal transduction [73,74]. In contrast, other studies suggest that the low molecular weight allows the EPSs to pass easily across cell membrane barrier, leading to suppressive functions such as cell cycle arrest [75]. In this regard, EPSR4 exhibited a molecular weight of 1.48 × 10^4^ g/mole and number average molecular weight of 1.12 × 10^4^ g/mole with sulfation of 48%. The observed anti- proliferative activity could be attributed to the unique chemical function groups such as sulphate [76,77], β-glycosidic linkages [77], protein molecules [78] and side chains [79].

Next, we explored the anti-inflammatory activity of EPSR4 by evaluating its inhibitory potency toward 5-LOX, COX-2 and hemolysis activities (Figure 11, Figure 12 and Figure 13). The inflammation process is initiated as a protective defense in response to various stimuli. However, persistent inflammation has been linked to various diseases, including neurological disorders, cancer, and cardiovascular diseases. Among different regulators of arachidonic acid metabolism, the over expression of 5-LOX and COX-2 has been associated with several inflammatory diseases [52]. Targeting of 5-LOX/COX-2 enzymes has been emphasized for anti-inflammatory drugs based on their ability to attenuate the production of leukotrienes and prostaglandin [53]. Toward this end, our results demonstrated that EPSR4 exhibit a significant inhibitory potency toward 5-LOX activity (IC_50_= 54.3 ± 2.1 µg /mL) compared to ibuprofen (IC_50_ of 1.5 ± 1.3 μg/mL) (Figure S4), while it showed moderate inhibitory activity toward COX-2 activity (IC_50_ > 125 µg/mL) compared to celecoxib (IC_50_ of 0.28 μg/mL) (Figure S5). Our findings were further affirmed by investigating the effect of EPSR4 on membrane stabilization. The results revealed that EPSR4 possesses a potent inhibitory activity toward hemolysis activity with IC_50_ of 112.2 ± 1.2 µg/mL, compared to indomethacin (IC_50_ of 17.02 ± 0.82 µg /mL) (Figure S6). These results indicate that EPSR4 possesses potent anti-inflammatory activity, which could be attributed to its high inhibitory activity toward 5-LOX enzyme, which regulates the expression of several cytokines and their related transcription factors [17,18]. Furthermore, the anti-inflammatory activity of EPSR4 could be associated with its antioxidant activity. Several studies have demonstrated that the exposure of macrophage cells to oxygen radicals or hydrogen peroxide radicals induces the expression of the COX-2 enzyme [17,18]. Several microbial metabolites showed the ability to trigger macrophages and modulate immunity to secrete cytokines and drive phagocytosis [80]. The EPS isolated from *Lactobacillus pentosus* LZ-R-17 demonstrated the ability to enhance the macrophage viability, improve phagocytosis and macrophage activation, and to promote the section of TNF- α, Il-1, IL-10, and IL-6 [27]. Furthermore, EPS extracted from *Bifidobacterium longum* BCRC 14634 exhibited considerable immunomodulatory action on macrophages, elevated IL-10 expression, and reduced the level of TNF-α [81]. In accordance with our findings, EL-Newary et al. showed that the BAEPS isolated from marine *B. amyloliquefaciens* 3MS exhibits a significant anti-inflammatory activity through targeting COX-2 activity, as well as scavenging capacity toward nitric oxide [82].

Finally, we have assessed the anti-Alzheimer activity by evaluating its inhibitory activity toward AChE activity. The AChE enzyme is highly abundant in the brain, neurons, and red blood cells and responsible for the hydrolysis of acetylcholine ester [83]. Under certain neurological conditions, the activity of the AChE enzyme and others in the cholinergic system is diminished. Accumulation of amyloid has been connected to the neural damage in the CNS and the Alzheimer’s disease pathogenesis. The hyperactivity of cholinergic has been linked to the metabolism of beta-amyloid precursor [84]. Accordingly, the clinical neuroprotective application of AChE inhibitors has been proposed for neurological disorder diseases, including Alzheimer’s disease, senile dementia, ataxia, Parkinson’s disease, and myasthenia gravis, based on its effect on the beta-amyloid metabolism [17,18]. Alternation of the AChE activity could restore the cholinergic balance by inhibiting Ach hydrolysis, which might minimize the progress of Alzheimer’s disease and enhance cognitive function [85]. However, there are some critical issues associated with finding novel AChE inhibitors for therapeutical application, including gastrointestinal troubles and bioavailability [86]. Our results showed that EPSR4 exhibits considerable inhibitory activity toward AChE activity with an IC_50_ of 786.38 µg/mL compared to Eserine with IC_50_ value of 0.09 µg/mL (Figure S7). Furthermore, epidemiological and experimental investigations demonstrated that inhibition of COX-2 activity attenuates the inflammatory process, which plays an important role in neurodegeneration associated with Alzheimer disease [87]. Accordingly, several studies have highlighted the clinical application of non-steroidal COX-2 inhibitors to delay the clinical expression of Alzheimer disease [17,18]. To this end, the potential activity of EPSR4 as selective anti-cyclooxygenase and its inhibitory activity against acetyl cholinesterase activity, along with its antioxidant properties, may offer EPSR4 as a valuable natural polysaccharide for treating and/or limiting Alzheimer disease. Our current research topic mainly includes the discovery of novel bioactive EPS and an exploration of their chemical structure and biological activity. We reported several analytical characterizations of the chemical structure but also screened for five different biological activities. Exploration of the chemical structure of ESPR4 included: chemical-based analyses (Uronic acid analysis, Sulphate analysis, and phenol-sulfuric acid analysis), spectroscopic analysis (FT-IF, UV-Vis), chromatographic analysis (HPGPC, HPLC), and morphological analysis (XRD, SEM, AFM). These analyses have revealed several critical structural features of the extracted EPSR4 and could predict the structure of EPSR4 (Figure 15). Furthermore, screening of the biological activity highlighted EPSR4 as a natural polysaccharide with potent biological activities that would be useful for the healthcare system.

## 4. Materials and Methods

### 4.1. Chemicals and Reagents

All solvents and chemicals used in the present study have been obtained from Sigma Aldrich Co. Ltd. (St. Louis, MO, USA). L-glutamine, HEPES buffer solution, RPMI-1640, DMEM, gentamycin, 0.25% Trypsin-EDTA, and fetal bovine serum were purchased from Lonza.

### 4.2. Sample Culture, Isolation and Identification of Bacterial Strains

The bacterial samples were obtained from marine sediment in the Red Sea. The bacteria were isolated using a media containing the following reagents: 20 g/L glucose, 1.0 g/L CaCO_3_, 0.8 g/L NH_4_NO_3_, 0.05 g/L KH_2_PO4, 0.6 g/L K_2_HPO_4_, 0.05 g/L MgSO_4_.7H_2_O, 0.1 g/L MnSO_4_. 4H_2_O, and 0.1 g/L yeast extract. These reagents were dissolved in 750 mL sea water and completed to 1000 mL [32]. The isolated bacterial strains were characterized according to the biochemical and morphological properties. The bacterial isolate was selected based on the strain which produced the maximum amount of EPSs with high antioxidant activity [33]. The identification of the bacterial isolate was confirmed by phylogenic analysis [34] using PCR analysis utilizing the 16S rRNA 5′-TCCGTAGGTGAACTTTGCGG-3′ primer and the reverse primer 5′-TCCTCCGCTTATTGATATGC-3′ [35]. The DNA sequence results were analyzed and compared to the GenBank database utilizing the BLAST program. The phylogenetic diagram was generated by aligning sequences with the greatest resemblance to the 16S rRNA sequences of the isolated bacterial. The 16S rRNA gene sequences of the bacteria was deposited in the nucleotide sequence database (https://www.ncbi.nlm.nih.gov/, accessed on 15 December 2021).

### 4.3. Production and Fractionation of EPS

Based on our screening for the bacterial strains, the bacterial isolate (AG4) showed to be the best bacterial strain which produced the maximum number of EPSs with high antioxidant activity. Accordingly, AG4 strain was chosen for the production of EPSs. The bacterial isolate AG4 was grown in a media containing the following reagents: 2 g/L yeast extract, 20 g/L sucrose, and 4 g/L peptone. The reagents were added to 750 mL sea water and the solution was diluted to 1 L [88]. After incubation, bacteria cells were removed by centrifugation at 4000 rpm at 4 °C for 30 min. Subsequently, TCA (10%) was added to remove protein and the resulting solution was then kept at 4 °C overnight. The solution was centrifuged again for 20 min at 5000 rpm. and the supernatant was separated. Finally, the supernatant was treated in NaOH solution until it was pH-adjusted to 7 [89]. The supernatant was treated with four liters of cold absolute ethanol, and the precipitate was recovered using a centrifuge. The precipitate was re-dissolved in water, and dialysis was achieved for 72 h against deionized water. Precipitation of the dialyzed solution was fractionally achieved with 1, 2, 3, and 4 volumes of ethanol, respectively [90].

### 4.4. Structural Characterization of EPSR4

#### 4.4.1. General Characterizations (Homogeneity, FT-IR, UV-Vis, Mw, PI)

The Fourier transform infrared spectrum (FTIR) spectra of EPSR4 was obtained utilizing 200 mg KBr and 2 mg of the sample [91]. The spectra were recorded employing the Bruker Vector 22 Spectrophotometer FTIR-UNIT. The average molecular weight (*Mw*) of EPSR4 was defined by High Performance Gel Permeation Chromatography (HPGPC, Agilent 1100 Series System, Hewlett-Packard, Germany) using water as eluent solvent and flow rate of 0.5 mL/min over GEL GMPWXL column. The Mw was detected using an Evaporative Light-scattering Detector (ELSD) with refractive index detection (*RI*) [92]. The polydispersity index (*PI*) was defined as the ratio between the molecular weight (Mw) and the number average of molecular weight (Mn) [93].

#### 4.4.2. Uronic Acid Analysis

The presence of uronic acid was determined using the method reported by Filisetti-Cozzi and Carpita [94]. This method is based on colorimetric determination of m-hydroxybiphenyl at 525 nm. Briefly, a sample of EPSR4 was treated with concentrated sulfuric acid (2 mL) and the resulting mixture was heated for 20 min at 100 °C. After the mixture was cooled to an ambient temperature, it was treated with m-hydroxydiphenyl (150 μL) and the mixture was incubated for 1 h at an ambient temperature. The absorption of the final mixture was recorded at 520 nm.

#### 4.4.3. Sulphate Analysis

The sulfate content of EPSR4 was evaluated following the reported method by Dodgson and Price [95]. Briefly, EPSR4 was eluted on SDSPAGE (7.5% *w*/*v*) and, subsequently, the obtained gel was treated with methylene blue (0.5% *w*/*v*) in acetic acid (3% *v*/*v*).

#### 4.4.4. Monosaccharide Composition Analysis

The monosaccharide composition analysis was determined following the methods reported by Liu et al. and Randall et al. [89,96]. Briefly, EPSR4 was treated with trifluoroacetic acid (2 M) for 2 h at 120 °C to fully hydrolyze the polysaccharide structure. The mixture was subsequently diluted with methanol and the solvents were removed under reduced pressure. The resultant residue was dissolved in water and analyzed on Aminex carbohydrate HP-87C column (300 × 7.8 mm) using water as eluent and flow rate of 0.5 mL/min (Agilate Pack, serics1, 200). Glucose (Glc), galactose (Gal), D-galacturonic acid (GalA), mannose (Man), and D-glucuronic acid (GlcA) were utilized as standards.

#### 4.4.5. Morphological Analysis

The crystalline phase of the extracted EPSR4 has been studied using the X-ray diffraction technique model Bruker D8 ADVANCE in theta/2 theta mode in the range (5–85) degrees utilizing Cu kα1 radiation of wavelength 1.5418 Å. The investigation of the EPSR4 surface morphology was performed using field emission scanning electron microscope model Quanta FEG-250 under high tension voltage of about 20 kV. Moreover, an atomic force microscope, AFM, (model: Bruker MLCT-MT-A) in a contact mode was utilized for exploring the surface nano-roughness of EPSR4.

### 4.5. Evaluation of Antioxidant Activity

#### 4.5.1. DPPH Assay

The antioxidant activity of EPSR4 (100, 300, 500, 1000, and 1500 g/mL) was assessed employing the DPPH assay. In this assay, the ability of EPSR4 to scavenge the DPPH free radical was evaluated at different concentrations over time intervals of 30, 60, 90, and 120 min. The assay was performed following the previously reported method by Gülcin et al. [50,97]. Briefly, DPPH solution (0.1 mM in ethanol) was added to a solution of EPSR4 in water at different concentrations (100, 300, 500, 1000, and 1500 ug/mL). The resulting mixture was vigorously vortexed and incubated in the dark. The absorption of the mixture was recorded at 517 nm at different time intervals 30, 60, 90, and 120 min. The ability of EPSR4 to scavenge DPPH free radical was assessed employing the following formula:

ability to scavenge DPPH free radical (%) = [(A_c_ − A_s_)/A_c_] × 100

where A_C_ the absorbance of the control sample; A_S_ the absorbance in the presence of EPSR4.

#### 4.5.2. Hydrogen Peroxide Scavenging (H_2_O_2_) Assay

The antioxidant activity of EPSR4 was evaluated by assessing its ability to scavenge hydrogen peroxide. The assay was performed following the previously reported protocol by Ruch et al. [98]. Briefly, EPSR4 was prepared at different concentrations (200, 400, 600, 800, 1000, and 1500 µg/mL) in 0.1 M phosphate buffer (pH = 7.4). The EPSR4 solutions were treated with a solution of hydrogen peroxide (43 mM in phosphate buffer). The resultant solutions were incubated in the dark at an ambient temperature and the absorption was recorded at 230 nm at different time intervals (15, 30, 45, and 60 min). The ability of EPSR4 to scavenge the hydrogen peroxide free radical was defined following the formula:

ability to scavenge H_2_O_2_ free radical (%) = [(A_c_ − A_s_)/A_c_] × 100.

where A_C_ the absorbance of the control sample; A_S_ the absorbance in the presence of EPSR4.

### 4.6. Assessment of Cytotoxic Effects Using Different Cell Line

A-549 (lung cancer), HepG-2 (hepatocellular carcinoma), and T-24 (bladder carcinoma) mammalian cell lines were obtained from VACSERA tissue culture unit. In a 96-well plate, the cells in 100 µL of growth medium and 1 × 10^4^ cells/well concentration were seeded. After 24 h of incubation, the cells were treated with EPSR4 at different concentrations (0, 3.9, 7.8, 15.6, 31.25, 62.5, 125, 250, 500 μg/mL in DMSO) or Cisplatin (0–100 μg/mL in DMSO), while the control cells were treated only with DMSO (max concentration 0.1%). After the plates were kept in a humidified incubator with 5% CO_2_ at 37 °C for 24 h, the number of viable cells was assessed [99,100,101,102]. Toward this, the media was extracted, and cells were treated with 1% crystal violet staining solution (crystal violet 0.5% *w*/*v*, methanol and distilled deionized water). After incubation for 30 min, the stain solution was separated, and the wells were washed with water. Finally, the wells were treated with 30% glacial acetic acid and the plates were shaken on a Microplate reader (TECAN, Inc., Männedorf, Switzerland). The absorbance of the wells was evaluated at a wavelength of 490 nm, utilizing the microplate reader (SunRise, TECAN, Inc., USA). All of these experiments were performed in triplicate. The percentage of cell viability was defined following the formula:

Cell viability (%) = [1 − (ODt/ODc)] × 100.

where ODt the mean of optical density for EPSR4 samples; ODc the mean of optical density for untreated cells (DMSO treated). The IC_50_ value was assessed at the concentration which causes cell viability of 50%, and it was assessed utilizing the Graphpad Prism (San Diego, CA. USA).

### 4.7. Assessment of the Anti-Inflammatory Activity

#### 4.7.1. Lipoxygenase (LOX) Inhibition In Vitro

The anti-inflammatory activity of EPSR4 was examined by evaluating the inhibitory activity of LOX enzyme. The inhibitory activity of EPSR4 toward the 5-LOX enzyme was assessed following the reported method by Granica et al. with minor modifications [57]. In this investigation, ibuprofen was used as the positive control drug. Briefly, LOX solution from soybean in borate buffer solution (1000 U/mL, pH 9) was mixed with EPSR4 at different concentrations (0.98–125 µg /mL in DMSO) or with Ibuprofen (0–125 µg/ mL in DMSO) for 15 min at ambient temperature. The obtained mixture was then treated with linoleic acid and the absorption of reaction was monitored by microplate reader (BIOTEK; Winooski, VT, USA) at 234 nm. The LOX inhibitory activity was assessed using the following formula:

LOX Inhibitory activity (%) = (1 − Ax/Ay) × 100

where, Ax the absorbance of tested samples; Ay the absorbance of control sample. The IC_50_ value was detected at the concentration that causes 50% inhibition of LOX enzymatic activity.

#### 4.7.2. Cyclooxygenase (COX2) Inhibition In Vitro

The anti-inflammatory activity of EPSR4 was evaluated by examining its inhibitory potency toward the COX2 enzyme at different concentrations (0.98–125-µg/mL in DMSO) compared to Celecoxib (0–31.25 µg/ mL in DMSO). This assay was based on the oxidation of the *N*,*N*,*N*,*N*-tetramethyl-p-phenylenediamine (TMPD) by arachidonic acid in the presence of COX2 enzyme (EC 1.14.99.1). The inhibitory activity of EPSR4 toward the COX-2 enzyme was assessed following the reported method by Petrovic and Murray and Amessis-Ouchemoukh et al. [55,56]. The inhibitory activity was monitored utilizing a microplate reader (BIOTEK; USA) by evaluating the absorption at 611 nm. Celecoxib was used as the reference drug for the evaluation of COX2 enzymatic activity. The COX2 inhibitory activity was evaluated following the formula:

COX2 Inhibitory activity (%) = (1 − Ax/Ay) ×100

where, Ax the absorbance in the presence of EPSR4 or Celecoxib; Ay the absorbance of control reaction. The IC_50_ was determined at 50% inhibition of COX2 enzymatic activity.

#### 4.7.3. Membrane Stabilization

The hemolysis inhibitory activity of ESPR4 was evaluated following the reported method by Shinde et al., based on erythrocyte hemolysis induced by hypotonic solution [58]. Briefly, the blood, obtained from rats using heparinized syringes, was washed with 10 mM sodium phosphate buffer (154 mM NaCl, pH 7.4) and subsequently centrifuged at 3000× *g* for 10 min to provide erythrocyte suspension. Then, 0.50 mL of erythrocyte suspension (RBCs) was treated with 10 mM sodium phosphate buffer containing 50 mM NaCl (5 mL, pH 7.4). The resulting mixture was treated with EPSR4 (7.8–1000-µg/mL in DMSO) or indomethacin (0–1000 µg/ mL in DMSO) and then incubated at ambient temperature for 10 min. Finally, the mixture was centrifuged at 3000× *g* for 10 min and the absorption of the supernatant was examined at 540 nm. The percentage of hemolysis inhibition was calculated according to the following formula [58].

% Hemolysis inhibition = 100 × {A1 − A2/A1}

where: A1 = Optical density of control sample; A2 = Optical density of EPSR4 or indomethacin. The IC_50_ value was determined at 50% RBCs hemolysis inhibition.

### 4.8. Acetyl Cholinesterase Inhibition Assay

The inhibitory activity of EPSR4 against acetyl cholinesterase activity was evaluated following the method reported by Ingkaninan with minor modifications [103]. Briefly, 500 μL of DTNB (3 mM), 100 μL of acetylthiocholine iodide (15 mM), 275 μL of Tris–HCl buffer (50 mM, pH 8), and 100 μL of distilled H_2_O (as a blank) or standard drug Eserine or EPSR4 at different concentrations (100, 250, 500, 750 and 1000 μg/mL) were placed in 1 mL cuvette. To the reaction cuvette, 25 μL of acetylcholinesterase enzyme solution in Tris-HCl buffer (containing 0.28 U mL^−1^). The absorption of the enzymatic reaction was examined at a wavelength of 405 nm. The percentage of inhibitory activity was defined by the following formulation:

{(control absorbance − sample absorbance)/control absorbance} × 100.

### 4.9. Statistical Analysis

All experiments were performed in triplicate. The results are presented as the mean ± standard deviation. Data were analyzed by using one-way ANOVA followed by the Tukey post hoc test. The significance of the data was determined by *p* value; *p* > 0.05 is considered non-significant and *p* < 0.05 significant. Statistical analysis was performed utilizing the software program Graphpad prism (GraphPad Software, San Diego, CA, USA, 2007) [104,105,106].

## 5. Conclusions

In this study, we isolated and characterized a novel exopolysaccharide EPSR4 from marine *Bacillus subtilis* strain AG4. The structure of isolated EPS was characterized by several analytical techniques which revealed that EPSR4 is a *β*-glycosidic sulphated heteropolysaccharide with a unique chemical composition. We explored and screened the biological activity of isolated EPSR4. Our findings demonstrated that EPSR4 exhibits strong scavenging activity toward both DPPH and hydrogen peroxide free radicals. Furthermore, EPSR4 showed considerable inhibitory activity toward the proliferation of both lung cancer and human hepatoma cell lines. Investigations of the anti-inflammatory activity revealed that EPSR4 has significant inhibitory activity toward 5-LOX activity, along with a moderate activity toward both hemolysis and COX-2 activities. Finally, the assessment of AChE activity indicated that EPSR4 could be a valuable natural therapeutic for Alzheimer disease. Taken together, this study identified EPSR4 as a natural exopolysaccharide which has unique and valuable biological activities. Further studies should be conducted to determine the exact chemical structure of EPSR4 and to further investigate the biocompatibility in vivo and the exact mode of action of EPSR4.

## Data Availability

The data presented in this study are openly available in DDBJ/EMBL/GenBank nucleotide sequence databases at https://www.ncbi.nlm.nih.gov/ (accessed on 15 December 2021), reference number GenBank: OL814951.

## Supplementary Materials

The following supporting information can be downloaded at: https://www.mdpi.com/article/10.3390/metabo12080715/s1, Figure S1: HPLC analysis of the EPSR4; Figure S2: The UV spectra of EPSR4 in the range 200–800 nm; Figure S3: The activity of ascorbic acid to scavenge the free radical of DPPH at different time intervals; Figure S4: The inhibitory activity of ibuprofen against 5-LOX enzymatic activity; Figure S5: The inhibitory activity of celecoxib against COX-2 enzymatic activity; Figure S6: The inhibitory activity of indomethacin against hemolysis activity (membrane stability); Figure S7: The inhibitory activity of Eserine against acetylcholinesterase activity.
