# Supplementary material for: Novel Exopolysaccharide from Marine Bacillus subtilis with Broad Potential Biological Activities: Insights into Antioxidant, Anti-Inflammatory, Cytotoxicity, and Anti-Alzheimer Activity
Source: Metabolites. 2022 Jul 31;12(8):715. doi: 10.3390/metabo12080715 (PMC9413097; doi:10.3390/metabo12080715)
Supplement: Supplementary file 1 [file metabolites-12-00715-s001.zip › metabolites-1786326-supplementary.pdf]

Supplementary Information

Novel Exopolysaccharide from Marine Bacillus subtilis with Broad Potential Biological Activities: Insights into Antioxidant, Anti-Inflammatory, Cytotoxicity, and Anti-Alzheimer Activity

Basel A. Abdel-Wahab [1,2], Hanaa F. Abd El-Kareem [3], Ahmad Alzamami [4], Cinderella A. Fahmy [5,6], Basem H. Elesawy [7], Maged Mostafa Mahmoud [8,9,10], Ahmed Ghareeb [11], Ahmad El Askary [12], Hebatallah H. Abo Nahas [13], Nashwah G. M. Attallah [14], Najla Altwaijry [14] and Essa M. Saied [15,16,*]

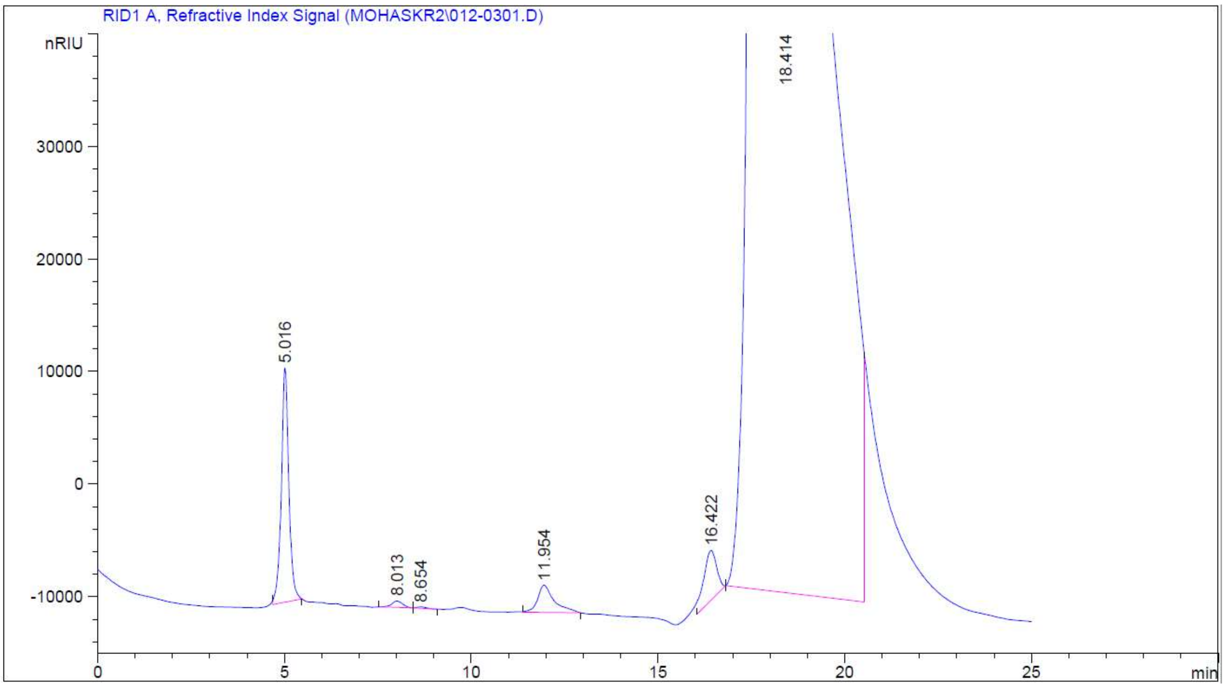

Figure S1: HPLC analysis of the EPSR4.

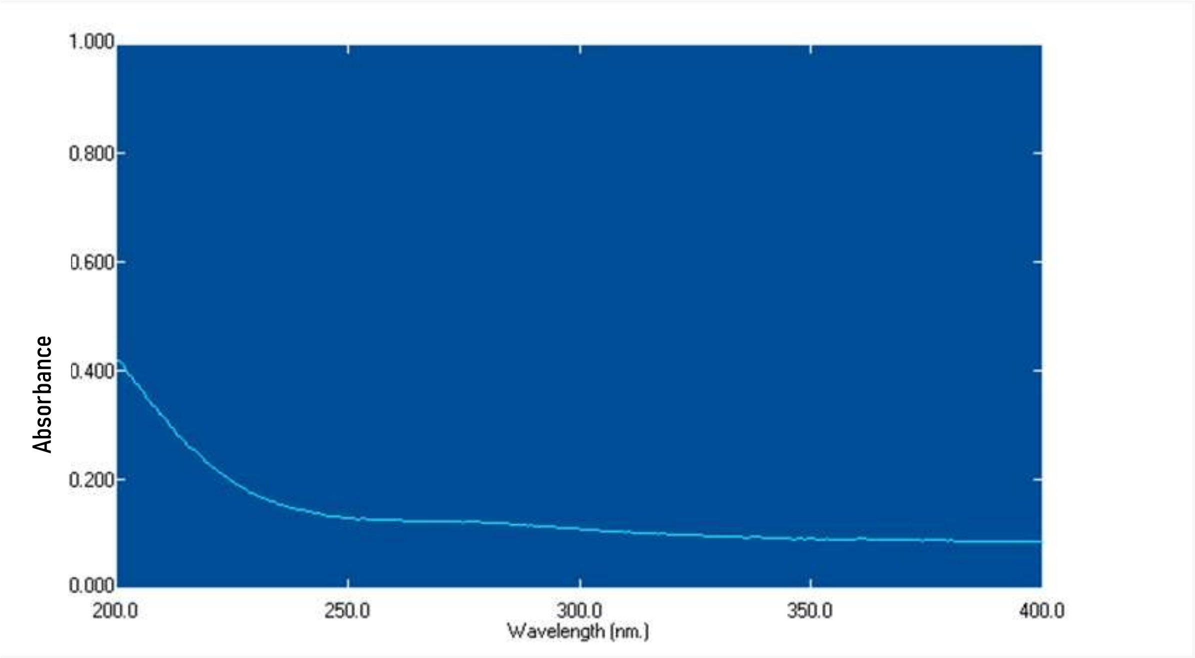

Figure S2: The UV spectra of EPSR4 in the range 200-800 nm.

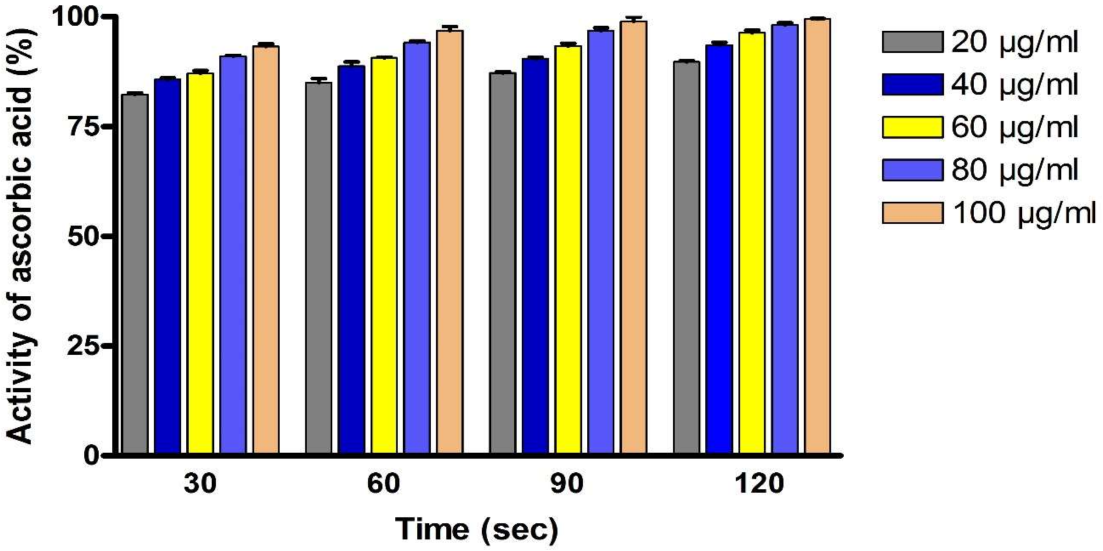

Figure S3: The activity of ascorbic acid to scavenge the free radical of DPPH at different time intervals**.**

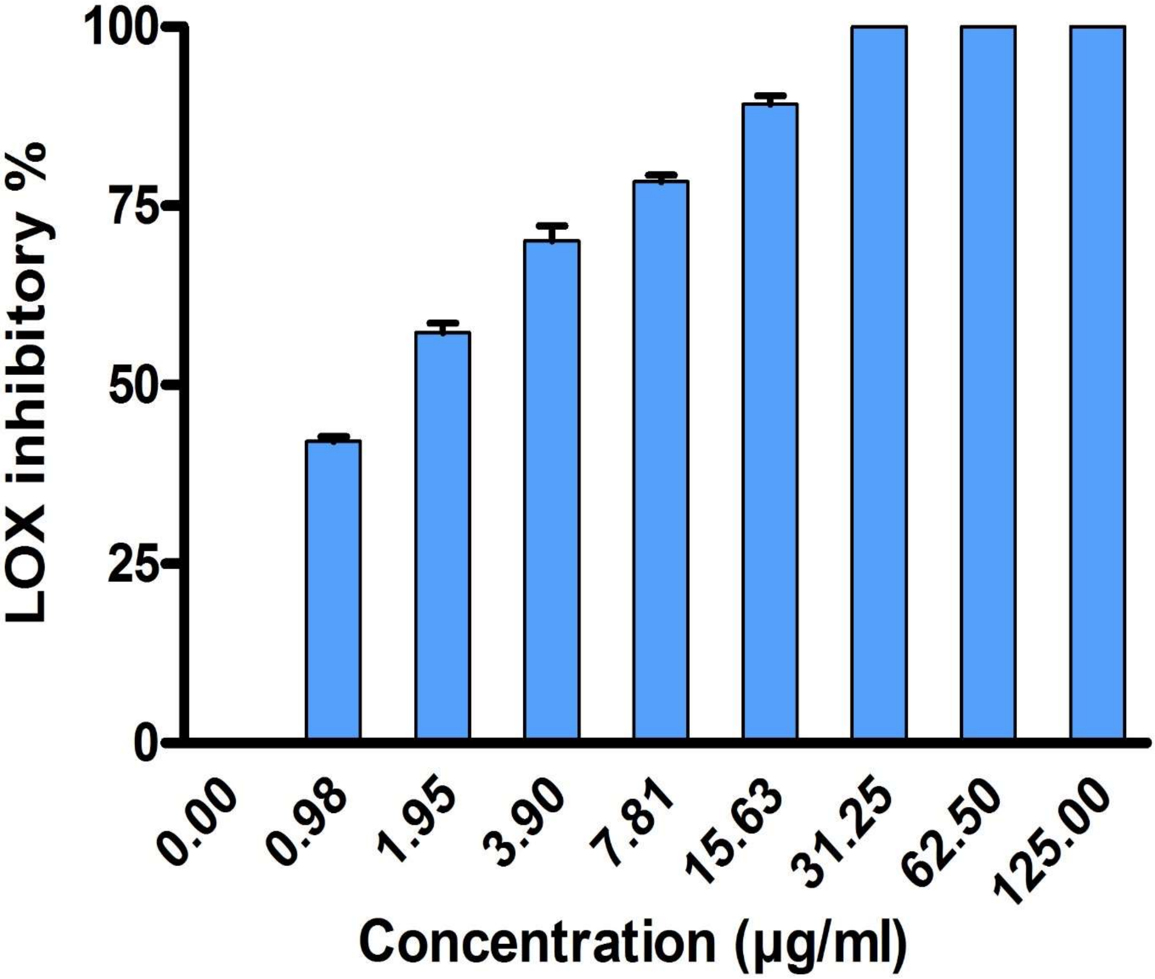

Figure S4: The inhibitory activity of ibuprofen against 5-LOX enzymatic activity.

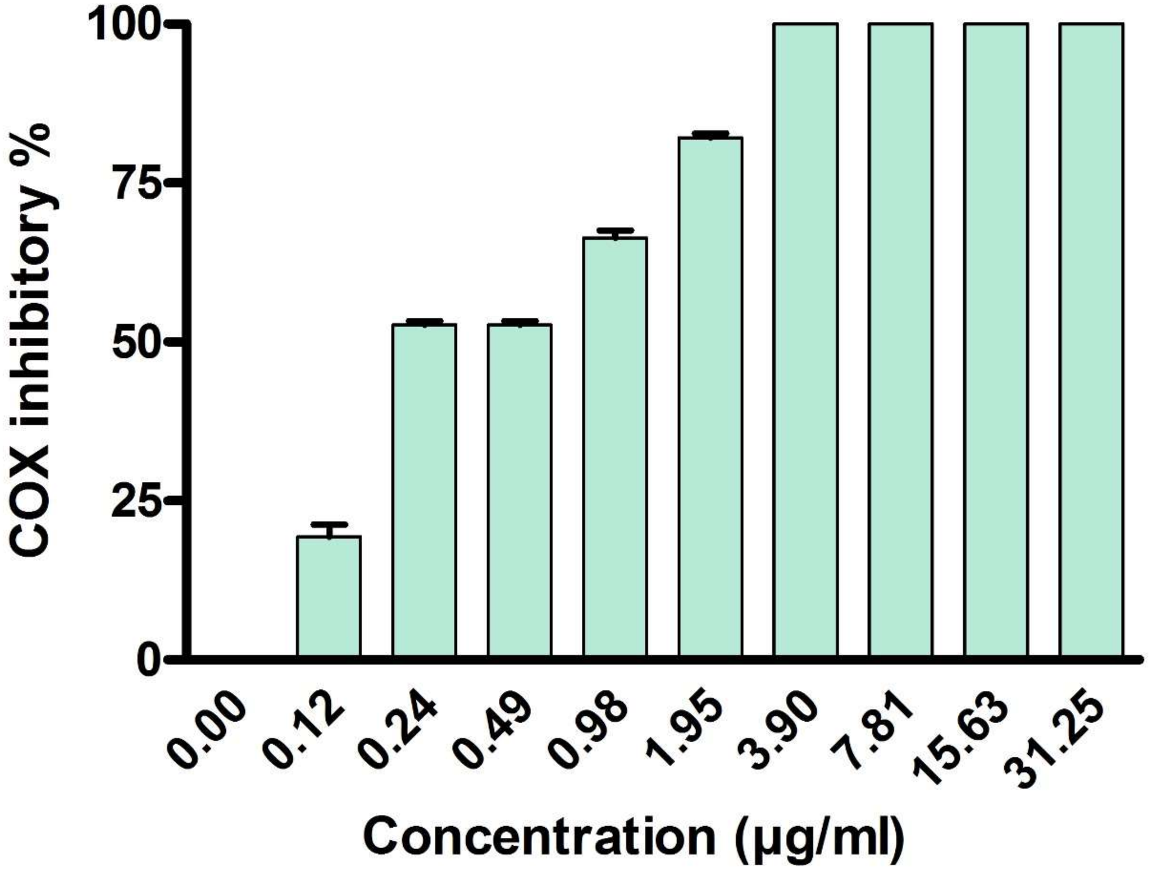

Figure S5: The inhibitory activity of celecoxib against COX-2 enzymatic activity.

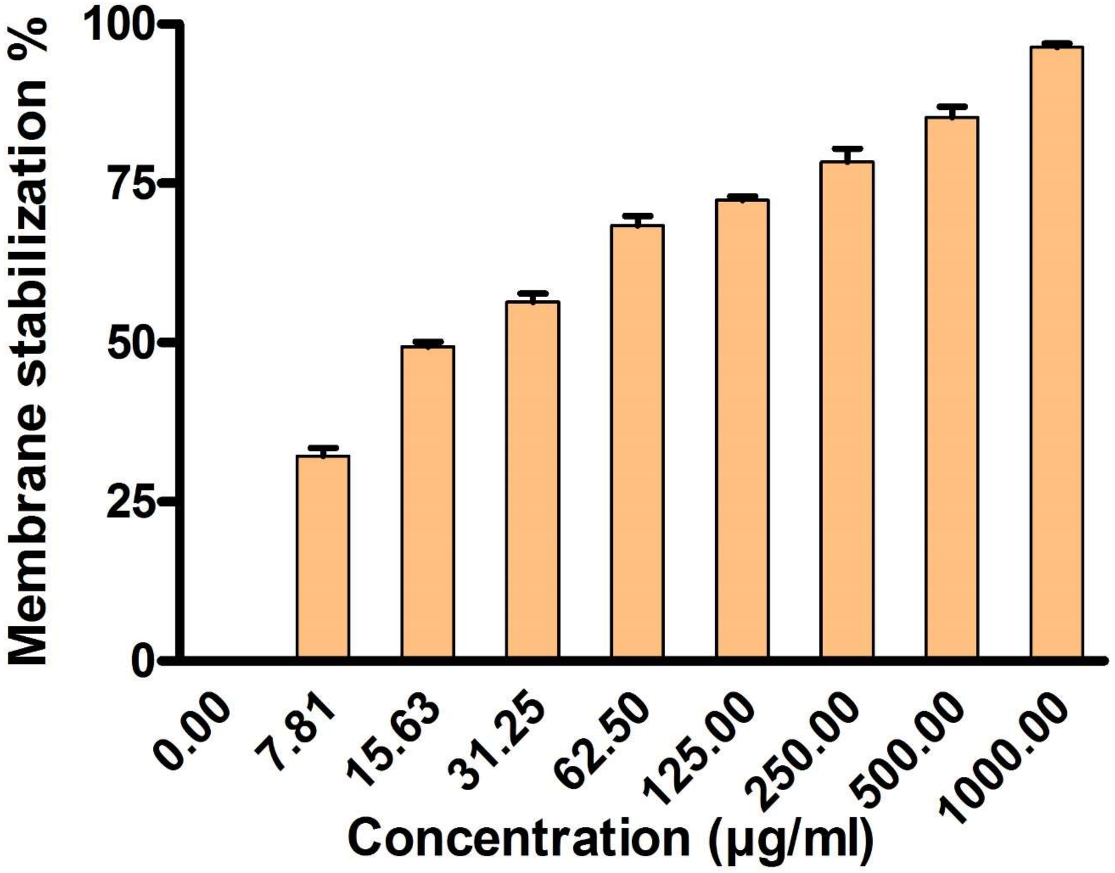

Figure S6: The inhibitory activity of indomethacin against hemolysis activity (membrane stability).

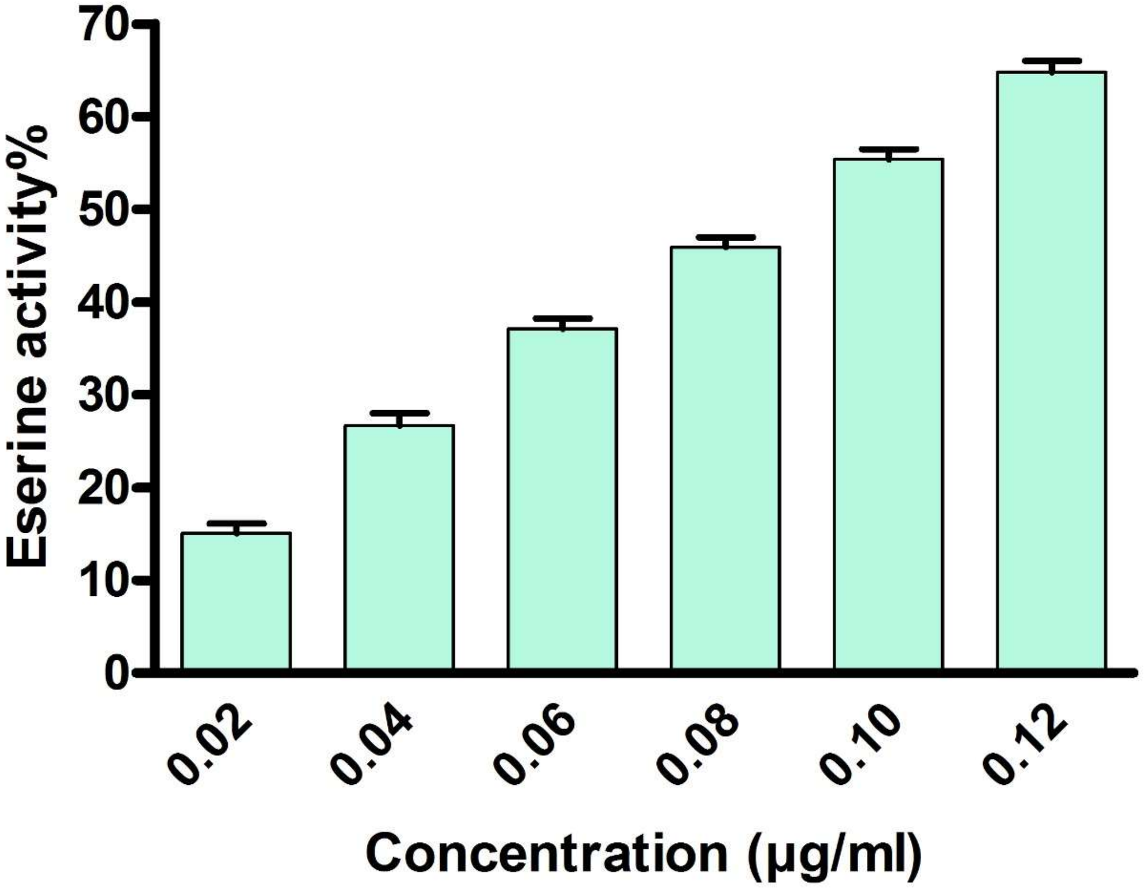

Figure S7: The inhibitory activity of Eserine against acetylcholinesterase activity.
